# Four Core Genotypes mouse model: localization of the *Sry* transgene and bioassay for testicular hormone levels

**DOI:** 10.1186/s13104-015-0986-2

**Published:** 2015-03-07

**Authors:** Yuichiro Itoh, Ryan Mackie, Kathy Kampf, Shelly Domadia, Judith D Brown, Rachel O’Neill, Arthur P Arnold

**Affiliations:** Department of Integrative Biology & Physiology, and Laboratory of Neuroendocrinology of the Brain Research Institute, University of California, 610 Charles E. Young Drive South, Los Angeles, CA USA; Institute for Systems Genomics and the Department of Allied Health Sciences, University of CT, Storrs, CT USA; Institute for Systems Genomics and the Department of Molecular and Cell Biology, University of Connecticut, Storrs, CT USA

**Keywords:** Four core genotypes, Sex chromosome, *Sry*, Mouse, Vectorette PCR, Inverted PCR, Anogenital distance, Transgene, Fluorescence *in situ* hybridization, Integration site

## Abstract

**Background:**

The “four core genotypes” (FCG) mouse model has emerged as a major model testing if sex differences in phenotypes are caused by sex chromosome complement (XX vs. XY) or gonadal hormones or both. The model involves deletion of the testis-determining gene *Sry* from the Y chromosome and insertion of an *Sry* transgene onto an autosome. It produces XX and XY mice with testes, and XX and XY mice with ovaries, so that XX and XY mice with the same type of gonad can be compared to assess phenotypic effects of sex chromosome complement in cells and tissues.

**Findings:**

We used PCR to amplify the *Sry* transgene and adjacent genomic sequences, to resolve the location of the *Sry* transgene to chromosome 3 and confirmed this location by fluorescence *in situ* hybridization (FISH) of the *Sry* construct to metaphase chromosomes. Using quantitative PCR, we estimate that 12–14 copies of the transgene were inserted. The anogenital distance (AGD) of FCG pups at 27–29 days after birth was not different in XX vs. XY males, or XX vs. XY females, suggesting that differences between XX and XY mice with the same type of gonad are not caused by difference in prenatal androgen levels.

**Conclusion:**

The *Sry* transgene in FCG mice is present in multiple copies at one locus on chromosome 3, which does not interrupt known genes. XX and XY mice with the same type of gonad do not show evidence of different androgen levels prenatally.

The four core genotypes (FCG) mouse model has the advantage of separating two major factors that cause phenotypic sex differences: sex chromosome complement (XX vs. XY) and gonadal hormones [1-10]. The FCG model was established by combining two mutations in the same mouse line: deletion of the *Sry* gene from the Y chromosome (producing the Y^−^ chromosome), and insertion of an *Sry* transgene onto an autosome [11,12]. Four genotypes are produced: XX mice with and without the *Sry* transgene, (XX*Sry*, XX), and XY^−^ mice with and without the *Sry* transgene (XY^−^*Sry*, XY^−^). Comparing XX and XY mice of the same gonadal type allows the measurement of the effect of sex chromosome complement (XX vs. XY) on traits in a similar hormonal environment. The *Sry* transgene has been used in over 60 primary literature articles (Table 1), and the FCG model is available commercially (Jackson Laboratory, Bar Harbor ME, strain 010905, B6.Cg-Tg(Sry)2Ei Sry < dl1Rlb>/ArnoJ). Here we report the location and number of copies of the *Sry* transgene.

**Table 1:** **Publications using the** ***Sry*** **transgene**

**Authors and years**	**Authors and years**
Abel et al., 2011 [13]	Markham et al., 2003 [14]
Barker et al., 2010 [15]	Mazeyrat et al., 2001 [16]
Bonthius et al., 2012 [17]	McPhie-Lalmansingh et al., 2008 [18]
Burgoyne et al., 2002 [19]	Moore et al., 2013 [20]
Caeiro et al., 2011 [21]	Ngun et al., 2014 [22]
Carruth et al., 2002 [23]	Palaszynski et al., 2005 [24]
Chen et al., 2008, 2009, 2012, 2013a, 2013b [25-29]	Park et al., 2008 [30]
Cocquet et al., 2009 [31]	Quinn et al., 2007 [32]
Corre et al., 2014 [33]	Reynard et al., 2009 [34]
Cox and Rissman, 2011 [35]	Robinson et al., 2011 [36]
Dadam et al., 2014 [37]	Sasidhar et al., 2012 [38]
De Vries et al., 2002 [1]	Scerbo et al., 2014 [39]
Durcova-Hills et al., 2004 [40]	Seney et al., 2013a, 2013b [41,42]
Ehlen et al., 2013 [43]	Seu et al., 2014 [44]
Ellis et al., 2005 [45]	Smith-Bouvier et al., 2008 [46]
Gatewood et al., 2006 [47]	Szot et al., 2003 [48]
Gioiosa et al., 2008a, 2008b [49,50]	Toure et al., 2004, 2005 [51,52]
Ishikawa et al., 2003 [53]	Van Nas et al., 2009 [54]
Ji et al., 2010 [55]	Vernet et al., 2011, 2012 [56,57]
Kopsida et al., 2013 [58]	Wagner et al., 2004 [59]
Kuljis et al., 2013 [60]	Ward and Burgoyne, 2006 [61]
Kuo et al., 2010 [62]	Wijchers et al., 2010 [63]
Li et al., 2014 [64]	Xu and Arnold, 2005 [65]
Liu et al., 2010 [66]	Xu et al., 2002, 2005a, 2005b, 2006, 2008a, 2008b [67-72]
Mahadevaiah et al., 1998 [12]	Yamauchi et al., 2010 [73]
Manwani et al., 2015 [74]	

An important issue is whether XX and XY FCG mice with the same type of gonad experience different levels of gonadal hormones, which therefore might confound the effects of sex chromosome complement (XX vs. XY). Previous studies have not detected differences in the levels of testosterone in XX vs. XY adult males, or in estradiol in XX vs. XY females groups [33,38,47,74]; R. Schafer, personal communication), but possible differences in levels of prenatal hormones have not been assessed. Here we measured anogenital distance postnatally. Because androgens secreted prenatally by the testes cause the AGD to be larger in mice with testes than in those with ovaries [75,76], AGD is considered an excellent bioassay for the prenatal levels of androgens. These effects of androgens cause permanent sex differences in AGD, and are classified as “organizational” effects of gonadal hormones.

## Methods

**Figure 1:**
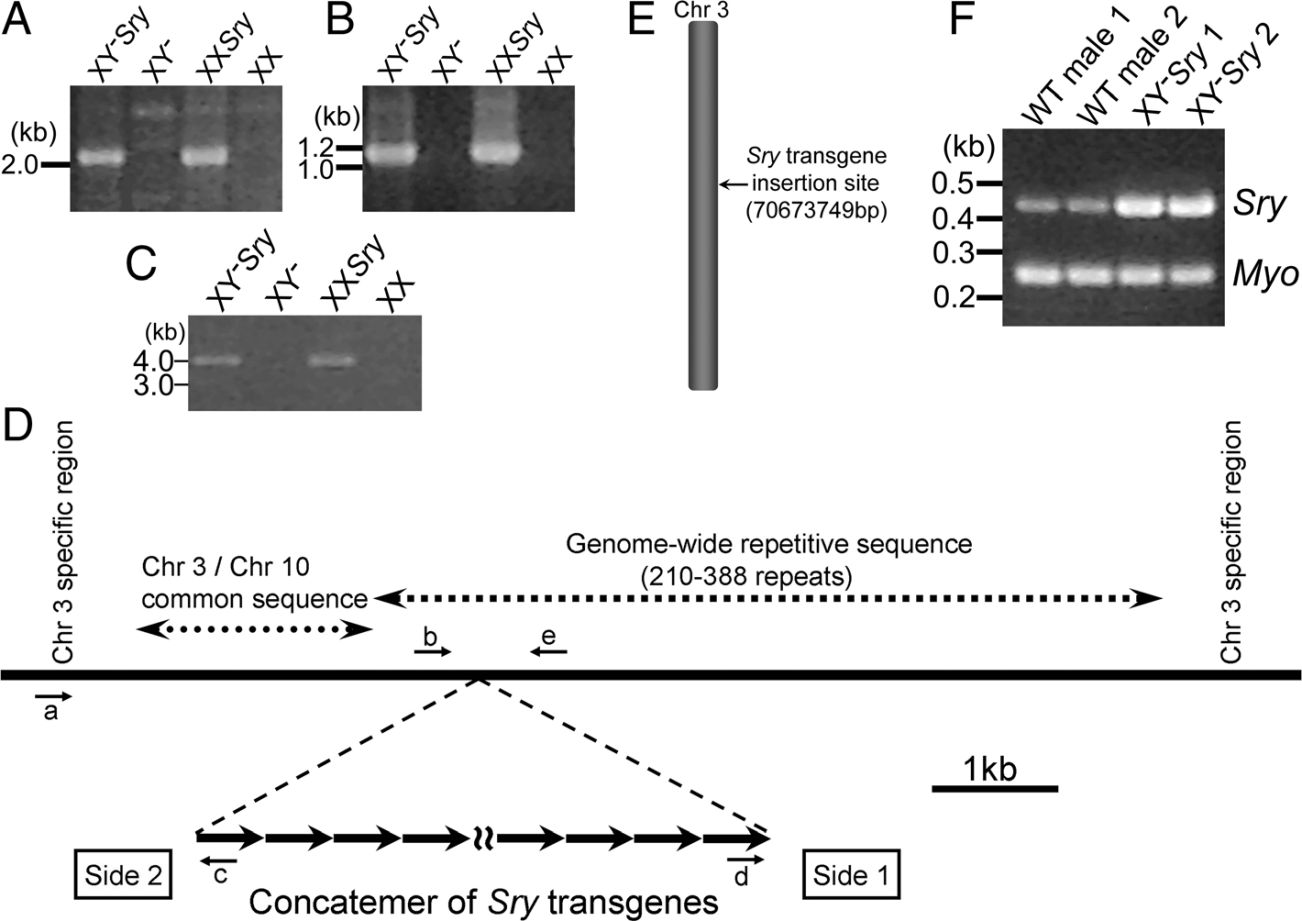
**Location of the** ***Sry*** **transgene in four core genotypes mouse model.** Samples were from XX and XY mice with testes (XX*Sry*, XY^−^ *Sry*) and XX and XY mice with ovaries (XX, XY^−^). Confirmation of transgene-genome boundary by PCR using transgene-specific primer *d* and Chr3 primer *e* **(A)** *,* with transgene-specific primer *c* and Chr3 primer *b* **(B)**, and with transgene-specific primer *c* and Chr3 primer *a* **(C)**. **(D)** The concatemer of *Sry* transgene is inserted into a repetitive sequence present genome-wide. **(E)** *Sry* transgene location on chromosome 3. **(F)** A visual estimate of the difference in copy number of *Sry* between wildtype and XY^−^ *Sry* genomic DNA in agarose gel.

To identify the *Sry* transgene location, we first screened the DNA sequences flanking the transgene using inverted PCR [77] and vectorette PCR [78]. Amplified PCR fragments of the boundaries were sequenced, and their specificities were confirmed by PCR using 6 and 10 pairs of transgene-specific and flanking region primers on each end, using DNA from C57BL/6 FCG mice as templates. PCR was carried out with MyTaq HS Red Mix (Bioline USA Inc.). The PCR reaction started at 94°C for 4 min before the cycling reaction of 35 cycles of 94°C for 45 sec/60°C for 30 sec/72°C for 1 min, and then followed by single reaction at 72°C for 7 min. The PCR reaction mixture was separated by 1.5% agarose gel electrophoresis in 1 x TAE at 80 V. The primers used in Figure 1 were: a) 5′-CCA TCT GGC CTA TGA TGG AT-3′ (chr 3), b) 5′-CCT GCA GAC ATT CTC TGT GC-3′ (chr 3), c) 5′-GCA AAG CTG AAC AAG CAA CA-3′ (*Sry* transgene). d) 5′-CCA GGA CCA GGC AAT TAT GT-3′ (*Sry* transgene), e) 5′-TAA ATG GAG GGA AGC TGG AA-3′ (chr 3). Boundary DNA sequences are deposited in Genbank (accession: KF959637).

To estimate the number of *Sry* copies integrated in the insertion site, we used quantitative PCR (standard curve method) to amplify *Sry* transgenes from genomic DNA. The quantitative PCR primers for *Sry* and control *beta*-*2 microglobulin (B2m)* were: *Sry* (5′-TTC CAG GAG GCA CAG AGA TT-3′, 5′-GCA GGC TGT AAA ATG CCA CT-3′), *B2m* (5′-AGG CCA AAA GCT CAC TCA AA-3′, 5′-GTG AGT TCT GGC TCC ACC AT-3′). We also confirmed the FCG vs. WT difference in copy number non-quantitatively and visually on agarose gels with PCR using other primers: *Sry* (5′- AGC CCT ACA GCC ACA TGA TA-3′, 5′- GTC TTG CCT GTA TGT GAT GG-3′), *myogenin* (5′- TTA CGT CCA TCG TGG ACA GCA T-3′, 5′- TGG GCT GGG TGT TAG TCT TAT-3′).

To evaluate the influence of the *Sry* transgene on genes in the vicinity of the transgene, we analyzed the FCG and WT liver microarray expression datasets (GSE13264, GSE13265) [54]. Those comparable datasets were from C57BL/6J background, using the same microarray platform in the same lab. One dataset allows measuring changes in gene expression caused by the *Sry* transgene in gonadectomized FCG mice (using a 2-way ANOVA with factors of sex chromosome complement (XX vs. XY) and *Sry* transgene (present vs. absent). The other dataset compares gonadectomized WT males and females, allowing measurement of the effects of the endogenous *Sry* gene on the Y chromosome (one-way ANOVA). The strain origin of the Y chromosome differed in the two datasets. We report both the p-values of the ANOVAs (non-stringent analysis without correction for multiple testing), as well as more conservative False Discovery Rate p-values [79] (Table 2).

**Table 2:** **Expression of Chr3 genes near the** ***Sry*** **transgene**

	**FCG**			**WT**			**Distance**
**Probe**	**ANOVA**	**FDR**	**MF**	**ANOVA**	**FDR**	**MF**	**(bp)**
*Lxn*	0.001	0.035	-0.08	0.549	0.998	0.02	-3215751
*Rarres1*	0.868	0.963	-0.01	0.678	0.999	-0.02	-3194863
*Mfsd1*	0.187	0.530	-0.04	0.339	0.951	-0.05	-3090981
*Schip1*	0.033	0.225	-0.04	0.806	0.999	-0.01	-2608947
*Schip1*	0.568	0.837	-0.01	0.615	0.999		-2608947
*Il12a*	0.310	0.656	-0.01	0.598	0.999	0.01	-1983105
*Trim59*	0.021	0.179	-0.09	0.160	0.832	0.03	-1638461
*Trim59*	0.950	0.985	0.00	0.623	0.999	0.02	-1638461
*Kpna4*	0.868	0.963	0.00	0.760	0.999	-0.01	-1606600
*Ppm1l*	0.034	0.230	0.08	0.002	0.417	0.06	-1356888
*Nmd3*	0.085	0.362	0.07	0.083	0.734	0.10	-951764
***Sry transgene***							0
*Slitrk3*	0.346	0.684	-0.02	0.728	0.999	0.02	2374377
*Bche*	0.060	0.306	0.10	0.931	0.999	-0.01	2962059
*Serpini2*	0.676	0.890	0.01	0.942	0.999	0.00	4568621
*Pdcd10*	0.231	0.580	0.03	0.308	0.938	0.04	4842741
*Pdcd10*	0.336	0.677	0.02	0.278	0.926	0.04	4842741
*Serpini1*	0.130	0.447	0.01	0.509	0.993	-0.02	4883798
*Fstl5*	0.917	0.978	0.00	0.831	0.999	0.01	5400521
*Rapgef2*	0.900	0.973	0.00	0.319	0.946	0.03	8388767
*Ppid*	0.002	0.056	0.14	0.674	0.999	0.02	8917593
*Etfdh*	0.024	0.191	0.09	0.987	0.999	0.00	8930039
*4930579G24Rik*	0.658	0.880	0.02	0.118	0.776	-0.07	8955330

The table shows ANOVA and False Discovery Rate (FDR) p-values of *Sry* effects on gene expression in liver of FCG mice (effect of *Sry* transgene) and of WT mice (effect of endogenous *Sry*). For several genes, p values for two different probes for the same gene are shown. MF is fractional mean difference between males (M, with *Sry*) and females (F, without *Sry*). For example, -0.08 means that F had about 8% higher expression than M. Distance is relative to the *Sry* transgene in FCG mice.

Metaphase chromosome spreads for FISH analysis were prepared from primary fibroblast cells cultured from tail tips. The *Sry* transgene plasmid construct was labeled with AF555 dUTP by nick-translation and hybridization was performed at 37°C in a humid chamber for 18–20 hours in the presence of 10 ug mouse Cot1 DNA (Invitrogen) and 9.4 mg salmon sperm DNA in Hybrisol VII (MP Biomedicals). Post-hybridization washes were 1× 2 minutes 2XSSC/0.3% NP40 at 68°C, 1 × 2 minutes 2XSSC/0.1% NP40 at 25°C. Images were captured using an Olympus AX-71 equipped with the Genus imaging software (Leica). For chromosome 3 (Chr3) painting, biotin-labeled Chromosome 3 Star*FISH© paint (Cambio) was used with the addition of a pre-annealing step prior to hybridization at 37°C for 90 minutes, followed by signal detection with fluoresceinated avidin.

Anogenital distance was measured in 34–44 C57BL/6 J FCG mouse pups per genotype, at 27–29 days after birth, using a caliper. A two-way ANOVA (factors of sex chromosome complement, XX vs. XY, and *Sry* (present vs. absent) was used to assess group differences. The investigator was blind to the genotype. Genotypes of FCG mice was determined by standard PCR genotyping methods using the primers: *Sry* (5′-AGC CCT ACA GCC ACA TGA TA-3′, 5′-GTC TTG CCT GTA TGT GAT GG-3′), Ymt (Y chromosome-specific sequence, 5′-CTG GAG CTC TAC AGT GAT GA-3′, 5′-CAG TTA CCA ATC AAC ACA TCA C-3′), and myogenin (5′-TTA CGT CCA TCG TGG ACA GCA T-3′, 5′-TGG GCT GGG TGT TAG TCT TAT-3′).

## Findings

The inverted PCR and vectorette PCR methods indicated that DNA sequences flanking the transgene represent part of a repetitive motif that is found at 210–388 genomic locations (http://www.ensembl.org, Release 73). Figure 1A and B show the transgene-specific PCR amplification between *Sry* transgene sequence and the surrounding repetitive sequence. These were not informative for mapping the transgene in the genome, but some DNA fragments from vectorette PCR suggested that the transgene was integrated into the motif within Chr3. This conclusion was confirmed by amplification with Chr3-specific primer *a* and transgene-specific primer *c* (Figure 1C and D). The *Sry* transgene integration site was at Chr3 70673749-70673824 bp (Figure 1E, based on Ensembl Release 73), and involved deletion of 74 bp of Chr3 during integration. The integration did not interrupt any known protein coding genes or pseudogenes (Table 3). The gene closest to the integration site is the Gm10780 pseudogene, 15 kb distant from the transgene.

**Table 3:** **Chr3 genes near the** ***Sry*** **transgene**

**Ensembl gene ID**	**Start (bp)**	**End (bp)**	**Gene name**
ENSMUSG00000087848	69685467	69685580	Gm25621
ENSMUSG00000068969	69716986	69717393	Rpl32-ps
ENSMUSG00000027787	69721985	69749042	Nmd3
ENSMUSG00000043461	69819538	69859896	Sptssb
ENSMUSG00000077366	69962315	69962445	Gm23484
ENSMUSG00000027788	70007613	70028708	Otol1
ENSMUSG00000089507	70228747	70228874	Gm23477
	70673749	70673824	(Sry transgene)
ENSMUSG00000074877	70689092	70689380	Gm10780
ENSMUSG00000097252	70772379	70807291	AC105155.1

To assess if the transgene affected gene expression nearby, we compared expression of 22 probes in liver in FCG mice with and without the transgene (Table 2). Most nearby genes showed no effect of the transgene. In a few cases, expression was affected by *Sry,* which could have been a local effect or one mediated by testicular secretions downstream of *Sry*. To control for hormonally-induced changes in gene expression, we compared expression of the same genes in WT males (with endogenous *Sry*) vs. females using published microarray gene profiling. Two genes, *Lxn* and *Ppid,* show evidence of regulation by the *Sry* transgene but not by WT *Sry*, based on conservative analysis. These are about 3 megabases or more from the transgene. Based on less stringent analysis, several other genes are candidates for those differentially expressed by the transgene vs. WT *Sry*. Further work is needed to determine if the transgene effects are found in different tissues and conditions, and are direct or indirect.

**Figure 2:**
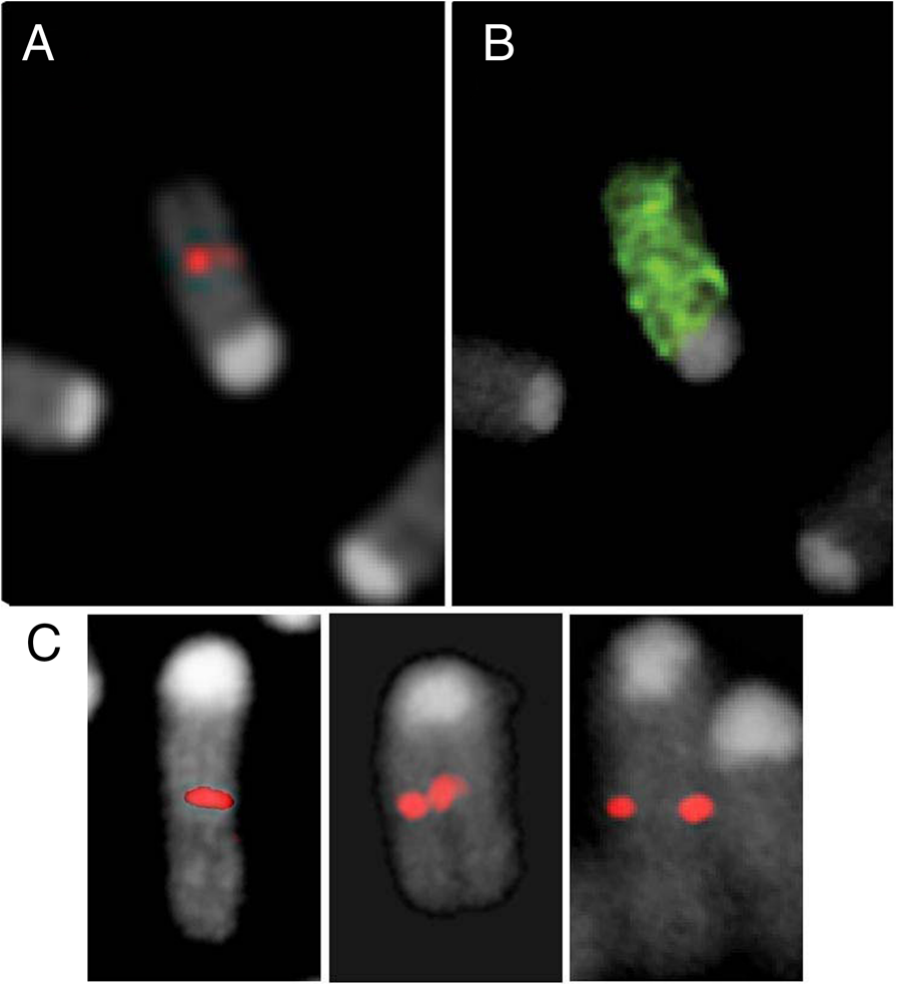
**FISH mapping of the** ***Sry*** **transgene to Chromosome 3.** The *Sry* transgene signal (**A**, red) and chromosome Chromosome 3 paint (**B**, green) hybridize to the same metaphase chromosome. **(C)** *Sry* transgene hybridization in three additional metaphase cells demonstrating its location with respect to the p- and q-arm ends.

The *Sry* transgene band in genomic DNA from FCG was stronger than in WT (Figure 1F), suggesting that the transgene was concatemerized during integration at this site. The number of copies of the transgene was estimated with quantitative genomic PCR (not shown) to be 12–14. The *Sry* transgene probe was co-localized with the Chr3 paint in metaphase spreads from FCG mice (Figure 2).

**Figure 3:**
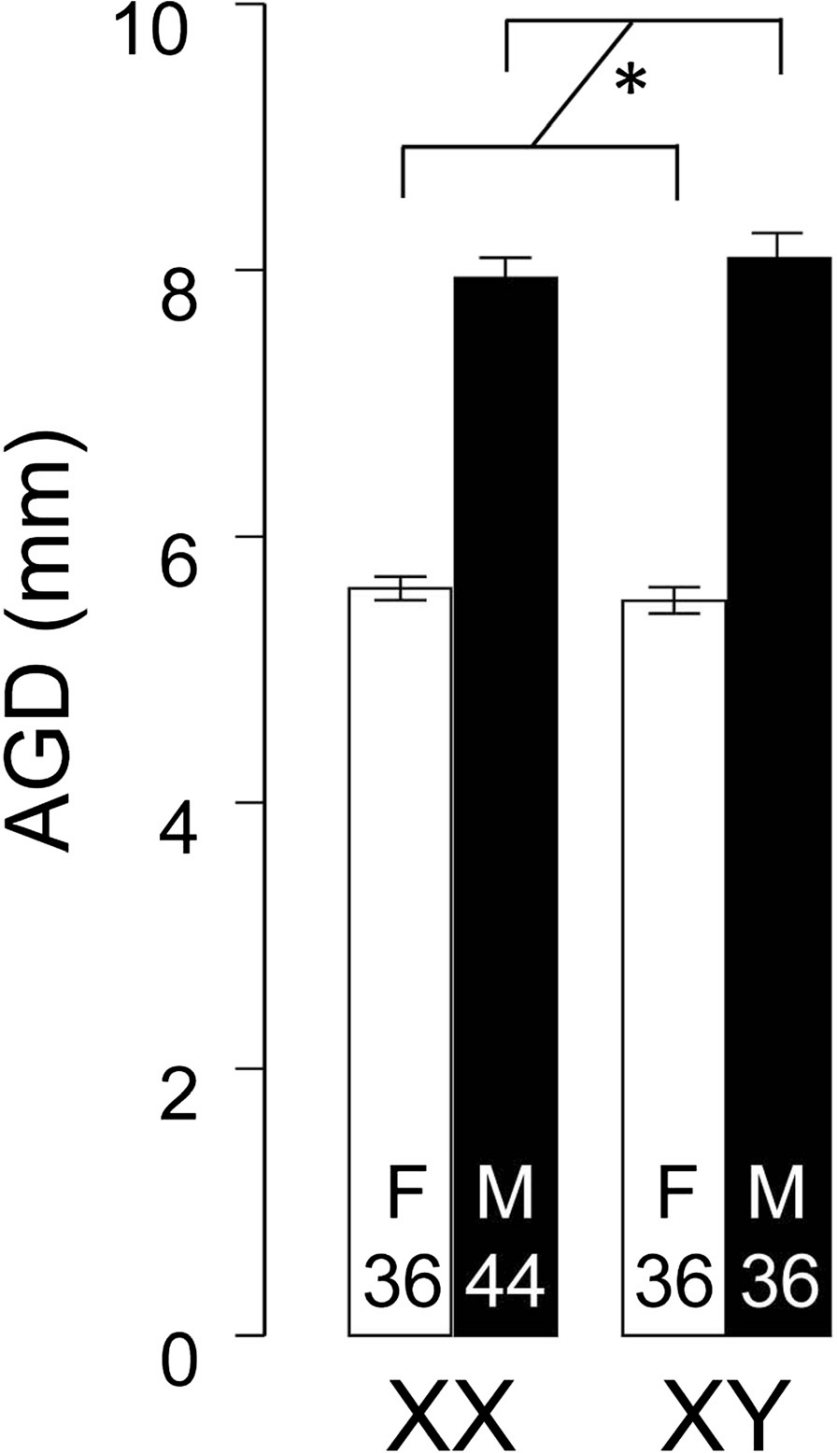
**Anogenital distance measurement of FCG mice.** Asterisk shows the significant effect of sex (two way ANOVA, p < 0.00001).

AGD was found to differ in mice with testes vs. ovaries (Figure 3), but not in XX and XY mice of the same gonadal sex (Figure 3). A two-way ANOVA showed a significant main effect of sex (F(1,146) = 223, p < 0.00001), but no effect of sex chromosome complement (XX vs. XY, F(1,146) = 0.03, p = 0.87) and no significant interaction (F(1,146) = 0.67, p = 0.42).

## Discussion

The goal of transgenic insertion is to achieve normal levels of expression of the transgene without influencing other genes because of interruption of coding or regulatory regions in the genome. Transgenic models are often useful even when this goal is not achieved in every respect. In the FCG model, mice with *Sry* are similar to WT males in numerous traits, but differ for a few other traits, including higher expression of *Sry*, indicating that the transgene effect is similar but not identical to endogenous *Sry* [1,74]. Here we show that insertion of *Sry* onto Chr3 does not disrupt any known coding sequence. Moreover, analysis of gene expression suggests that two genes near *Sry* may also be altered by the transgenic insertion, as judged by expression levels in liver. Further analysis is required to determine if local genes are affected by the transgene. The FCG model has the advantage of comparing the effects of sex chromosome complement (XX vs. XY) in mice with and without the transgene. The FCG model has been useful for discovering numerous traits that are influenced by sex chromosome complement, which are independent of the presence of the transgene or have been confirmed by analysis of non-transgenic mouse models that vary sex chromosome complement [25-28,64,80]. The concatemeric insertion of 12–14 copies of a transgene at one site is not unexpected, and in the present case is associated with higher than normal expression of *Sry* in FCG than WT mice [74].

The greater AGD in mice with testes is expected from previous studies that demonstrate that AGD is influenced by the level of prenatal androgens. The present data offer no support for the hypothesis that the levels of androgens secreted prenatally, when AGD is determined, differ in XX and XY mice with the same type of gonad. For example, there was no masculinization of AGD of XY females relative to XX females. That result argues against the idea that XX vs. XY differences observed in numerous tissues are a result of differences in levels of prenatal androgens.

The present results contribute to the understanding of the FCG model which is used increasingly to discriminate effects of sex chromosome complement and gonadal effects on sexually dimorphic non-gonadal phenotypes (Table 1).

## Abbreviations

FCG: Four core genotypes
AGD: Anogenital distance
FISH: Fluorescence *in situ* hybridization, WT: Wild type
